# Orthopedic surgeons’ knowledge regarding risk of radiation exposition: a survey analysis

**DOI:** 10.1051/sicotj/2017008

**Published:** 2017-04-07

**Authors:** Nejat Tunçer, Ersin Kuyucu, Şafak Sayar, Gökhan Polat, İrem Erdil, İbrahim Tuncay

**Affiliations:** 1 Bezmi-Alem University, Orthopedics and Traumatology Istanbul Turkey; 2 Medipol University, Orthopedics and Traumatology Istanbul Turkey; 3 Istanbul University, Orthopedics and Traumatology Istanbul Turkey; 4 Bakırköy Sadi Konuk Education and Training Hospital, Radiology Department Istanbul Turkey

**Keywords:** Orthopedics, Fluoroscopy, Scatter radiation, Radiation hazards, Radiation protection

## Abstract

*Introduction*: The purpose of this study is to evaluate the knowledge levels of orthopedic surgeons working in Turkey about the uses and possible risks of fluoroscopy and assess methods for preventing radiation damage.

*Methods*: A questionnaire with a total of 12 questions was sent to 1121 orthopedic surgeons working in Turkey. The questionnaire evaluated participants’ knowledge about the uses and risks of fluoroscopy and methods for preventing damage. One thousand and twenty-four orthopedic surgeons were found to be suitable for inclusion in the study. The effects of fluoroscopy on patients were not assessed in our study.

*Results*: The data obtained were statistically evaluated. Of the surveyed surgeons, 313 (30%) had used fluoroscopy in over 50% of their operations. The average number of fluoroscopy shots per case was 54.5. A lead apron was the most commonly used (88%) protection from the harmful effects of radiation. Fluoroscopy shots were performed with the help of operating room personnel (86%). A dosimeter was used 5% of the time.

*Conclusion*: According to the survey results, the need for fluoroscopy was very high in orthopedic surgery. However, orthopedic surgeons have inadequate knowledge about the uses and risks of fluoroscopy and methods for preventing damage. Therefore, we believe that training on this topic should be provided to all orthopedic surgeons.

## Introduction

Today, technological methods that cause minimal damage to soft tissue and reduce morbidity have become widespread, and minimally invasive surgical procedures have gradually increased in popularity. Indirect visualization of anatomy is one of the fundamental requirements in varied orthopedic approaches as in trauma, reconstructive and pediatric surgery. Although fluoroscopic imaging is a convenient method for obtaining such indirect views, operating with X-rays and exposing the patient to radiation for every image are obvious disadvantages [1, 2]. The harmful effects of radiation on the body are well known. Besides that, low dose radiation exposition may be a cumulative affect on the body. Surgeons are potentially at cancer risk due to recurrent fluoroscopic surgeries. However, precautions can be taken to minimize these side effects [3].

The aim of this study is to investigate the ratio of the fluoroscopic requirements in orthopedic surgery in Turkey. We also aimed to evaluate orthopedic surgeons’ knowledge levels about fluoroscopy precautions and preferred precautionary methods.

We hypothesize that most of the surgeons do not have adequate knowledge about fluoroscopy use and radiation protection.

## Methods

For this study, 1121 orthopedic surgeons actively working in Turkey were contacted by electronic mail or phone. All surgeons are still working in education or state hospitals and also have at least two-year experience in Turkey. They were invited to participate in a survey with a total of 12 questions (Table 1). Orthopedic residents, specialists, assistant professors, associate professors, and professors were included in the study. Ninety-seven surgeons who agreed to participate in the study were excluded because there was no fluoroscopy device in the hospital where they worked. Of the 3500 surgeons currently working in Turkey, 1024 (approximately 1/3) were included in the study. The remaining 1024 orthopedic surgeons (35 professors, 67 associate professors, 47 assistant professors, 362 specialists, and 513 residents) who completed the survey were included in the study.

**Table 1.** Questionnaire.

What is your title at the clinic where you are working? Professor Associate professor Assistant professor Specialist doctor Assistant doctor How many days per week do you have the opportunity to do surgery? 1 2 3 4 5 How many operations do you perform on average when you are in theatre? 1–2 3–4 5–6 More than 6 In how many of your operation do you need fluoroscopy? All the More than 50% 25–50% Less than 25% The average number of times you shoot in last five operations in which you used fluoroscopy? >10 10–50 50–100 <100 Do you know how the fluoroscopy device operates and how to use it? I know very well I know well I know less I do not know Who do you get help from when using fluoroscopy? Radiology technician Operating room personnel Medical company employee Assistant doctor Do you know the dose of radiation received with standard AP hip imaging? Yes No Have you read any literature on fluoroscopy? Yes No What do you use to protect yourself from radiation during fluoroscopic use? The lead aprons Thyroid protector Gonad protector Protective eyewear, glove I do not use anything Have you experienced any of the following complaints in the days that you use fluoroscopy? Headache Eye pain Nausea Fatigue I did not have any complaints Do you use a dosimeter? Yes No

The first part of the questionnaire included questions about the frequency of operating and the use of fluoroscopy. In the second part, doctors’ knowledge levels about fluoroscopy were examined by asking the average amount of radiation to which a patient is exposed during fluoroscopic imaging of the hip. This question was open-ended rather than multiple choices. In the third part of the survey, doctors were asked about their methods of protection from radiation.

## Results

Of the 1024 participants, 513 (50%) were residents, 362 (35%) were specialist doctors, 67 (6%) were associate professors, 47 (4%) were assistant professors, and 35 (3%) were professors. The average number of operating days per week was 1.7 (1–4) for all participants. Participants performed an average of 2.2 surgeries on operating days (1–5). While 635 (62%) participants used fluoroscopy in 25–50% of their surgeries, 313 (30%) participants stated that they needed fluoroscopy in over 50% of their surgeries. Seventy-six (8%) participants used fluoroscopy in less than 25% of their surgeries.

Surgeons who perform sports-related surgery, arthroplasty, and hand surgeries need less fluoroscopic images than trauma and reconstruction surgeons who expose the maximum fluoroscopy shots.

In minimally invasive trauma surgery, surgeons expose themselves to a higher dose of radiation than in open operations as expected. Surgeons need images only for reduction and implant length check in open surgeries. Intraarticular and metaphyseal fracture operations carry more risk than diaphysis fractures for radiation exposure.

Fluoroscopy is not routinely used in any phase of arthroscopy and soft tissue surgeries, hence surgeons who perform this type of surgery may be safe.

Participants used an average of 54.5 fluoroscopy shots in each surgery, and an inverse relationship was found between experience and the number of fluoroscopy shots. Assistant doctors used an average of 62.4 fluoroscopic shots per case; this number was 34.6 for professors, and the difference was statistically significant (*p* < 0.05).

Although 97.4% of the surgeons participating in the study said that they know how to use fluoroscopy devices, only nine of 1024 participants knew the amount of radiation dose received at standard antero-posterior (AP) imaging (0.8%). Almost all of the participants (99.2%) did not know the amount of radiation that a patient is exposed to during fluoroscopy. Only 87 of 1024 (8.5%) surgeons have read at least one article regarding fluoroscopy. There was no relation between the participants’ level of experience and this knowledge (*p* > 0.5).

The most commonly used protection methods were lead aprons (85%) and thyroid protectors (70%). Gonad protectors (30%), and glasses and gloves (5%) were used to a lesser extent.

Seven hundred twenty-one of 1024 (41%) surgeons complained about only headache, 279 of 1024 (27%) had both headache and fatigue, and 375 of 1024 (36%) had no complaints after fluoroscopy use.

Sixty-three percent of orthopedic surgeons get help from operating room personnel, only 22% get help from radiology technicians, and 15% of the participants get help from medical company employees and resident doctors. Only 5% of surgeons use dosimeters in operations.

Table 1 shows the contents of the questionnaire.

## Conclusion

In this study, we investigated the knowledge levels of orthopedic surgeons working in Turkey about fluoroscopic imaging as it is frequently used in orthopedics. However, operating with an X-ray can cause some acute, and chronic problems. This study also investigated the safety measures employed by orthopedic surgeons during the use of fluoroscopy.

Since radiography was discovered by Wilhelm Roentgen in 1895, scientists have searched for ways to create faster and brighter X-ray images, and this led to the introduction of the fluoroscopy device. Although it is superior to conventional X-ray in that results are obtained more quickly and the image is available on the screen during irradiation, the most significant disadvantage of fluoroscopy is that both the patient and examiner are exposed to more radiation than with conventional X-ray. However, all procedures can be set from the control panel in monitor-equipped devices, which have been developed in recent years, protecting employees from the direct effects of radiation.

Previous studies [4–6] documented that the amount of occupational radiation exposure for surgeons who used C-arm fluoroscopy was below the safe limit determined by the International Commission on Radiation Protection (IRCP). However, reliable and accurate data on this question is not available because many surgeons in these studies did not use a dosimeter.

The maximum annual doses of occupational exposure to radiation were defined by the Radiation Safety and Regulations published in the official newspaper of the Republic of Turkey on March 24, 2000 [7]. According to these regulations, the use of dosimeter is mandatory for individuals exposed to a dose of more than 6 mSv annually. In this study, only 10% of orthopedic surgeons questioned used a dosimeter (Question 12). The annual safe exposure for surgeons is not known because many surgeons do not use a dosimeter. Increased use of dosimeters would help determine appropriate levels of exposure, and individuals may also limit their use of fluoroscopy to prevent overdosing.

Fluoroscopy may have dose-dependent (deterministic) and dose-independent (stochastic) harmful effects on patients and employees [8–10]. The radiation dose per exposure has increased significantly, and dose-dependent effects, especially those incurred from interventional examinations, have received more attention. Dose-dependent and -independent effects of ionizing radiation are noticeable in the short and long term. Its short-term effects include headaches, severe fatigue, dermatitis, cataracts, and irritable colon, and the long-term effects include gene mutation and cancer. It was determined that 95% of the doctors who participated in the study have experienced headaches and fatigue at least once after surgeries using intense fluoroscopy (Question 11).

Radiation is definitely known to cause cancer, and radiation-induced cancers constitute 2% of all cancers [11, 12]. Although it has been demonstrated that the radiation in normal doses of fluoroscopy does not cause skin cancer, exposure to high doses of radiation such as radiotherapy is known to cause skin cancer [13–15]. Therefore, incorrect or excessive use of fluoroscopy can lead to exposure to high doses of radiation, which together with inadequate protection can significantly increase the risk of cancer. There was no cancer among the doctors participating in the study, but it should be noted that 85% of participating doctors were residents or specialist doctors at the beginning of their careers.

Orthopedic surgeons are often exposed to scatter radiation, not direct radiation, during surgery. The harmful effects of radiation are minimized by the use of a lead apron and devices to protect the gonads and thyroid gland. Studies have shown that the use of proper protection reduces a doctor’s radiation exposure by 90% [8, 15]. One challenge in this study is that only lead aprons and thyroid protectors were used (Question 10). Although doctors’ use of protection varied according to the type of operation, protective gloves were not used frequently, even though the surgeon’s hands were exposed to the most radiation during surgery. Moreover, only a small percentage of surgeons in this study used protective eyewear (Question 10).

One of the most important findings of this study was most surgeons did not have adequate technical knowledge regarding the use of fluoroscopy devices. The majority of participating orthopedic surgeons (75%) had not read any literature on fluoroscopy (Question 9). Although almost all of the participants have claimed to know how a fluoroscopy device operates and used fluoroscopy devices for some period during their training, they did not have adequate knowledge of radiation doses (Questions 6–8). Exposure to high doses of radiation cannot be prevented when surgeons do not know how many doses or exposures are necessary to obtain sufficient images of adequate quality. This problem cannot be solved by leaving the work of dose adjustment only to technicians. Moreover, this study found that 63% of surgeons get help from radiology technicians who have adequate technical knowledge (Question 7). This may lead to an unnecessary increase in the number of fluoroscopy shots and the amount of radiation released into the environment. Orthopedic assistants should be educated about the use of fluoroscopy, appropriate dosage for different regions of the body, and injection techniques for various types of patients in order to eliminate this problem. Better training in fluoroscopy can reduce operation time, unnecessary overdosing of patients, and indirect radiation exposure for surgeons.

The use of fluoroscopy and radiation exposure is an important issue that can affect the lives of orthopedic surgeons and not only their patients. This study is the first large study we have found on the use of fluoroscopy by orthopedic surgeons in Turkey.

One of the limitations of this study is that the majority of participants were in the early part of their careers and relatively inexperienced. Data on the long-term effects of fluoroscopy and the possible development of malignancies can be obtained by further studies including participants who have been working longer.

Devices and instruction manuals associated with the use of fluoroscopy have changed and evolved. Orthopedic surgeons can more efficiently utilize fluoroscopy and protect themselves properly from radiation by following current developments and regularly obtaining up-to-date information.

## Conflict of interest

All the authors declare that they have no conflict of interest.
